# CDDO-imidazolide ameliorates sepsis-induced ARDS by enhancing mitophagy via the Nrf2 pathway to prohibit alveolar macrophage pyroptosis and HMGB1 release: CDDO-Im ameliorates sepsis-induced ARDS

**DOI:** 10.3724/abbs.2025092

**Published:** 2025-07-02

**Authors:** Yajing Liu, Pengcheng Ye, Cijun Tang, Meiru Jiang, Yiru Shen, Xiangrui Wang, Lei Hou, Yupeng Zhao

**Affiliations:** 1 Department of Anesthesiology and Critical Care Medicine Shanghai East Hospital Tongji University School of Medicine Shanghai 200120 China; 2 Jinzhou Medical University Jinzhou 121001 China; 3 Department of Anesthesiology Tongren Hospital Shanghai Jiao Tong University School of Medicine Shanghai 200336 China; 4 Department of Anesthesiology Shanghai General Hospital Shanghai Jiao Tong University School of Medicine Shanghai 200080 China

**Keywords:** CDDO-imidazolide, pyroptosis, mitophagy, Nrf2, HMGB1

## Abstract

Accumulating evidence suggests that NLRP3-mediated alveolar macrophage (AM) pyroptosis and subsequent high mobility group box protein 1 (HMGB1) secretion play significant roles in the pathogenesis of acute respiratory distress syndrome (ARDS). Nrf2 has been shown to be individually involved in regulating pyroptosis. In this study, we investigate the ability of CDDO-imidazolide, a potent Nrf2 activator, to regulate AM pyroptosis and HMGB1 secretion in sepsis-associated ARDS, along with its underlying mechanism. The *in vitro* alveolar macrophage (AM) pyroptosis model, established by stimulating J774A.1 cells with LPS and ATP, was treated with CDDO-imidazolide or utilized *Nrf2*-knockout cells. The mice are intraperitoneally administered with CDDO-imidazolide before the *in vivo* sepsis-associated ARDS model is constructed via caecal ligation perforation and the Nrf2 inhibitor, ML385. *In vitro* studies reveal that the use of 3-MA to prohibit PINK1/Parkin-dependent mitophagy aggravates NLRP3-mediated pyroptosis and HMGB1 release in J774A.1 cells via LPS and ATP exposure. CDDO-imidazolide also significantly prevents NLRP3-mediated pyroptosis and HMGB1 release to increase PINK1/Parkin-dependent mitophagy, but these effects are not detected in *Nrf2*-knockout macrophages. Most importantly, CDDO-imidazolide significantly alleviates NLRP3 inflammasome protein expression in the lung tissues of septic mice and HMGB1 protein levels in the serum and bronchoalveolar lavage fluid (BALF), which can be reversed by ML385. Taken together, our results demonstrate that CDDO-imidazolide prominently protects the lungs by promoting Nrf2 activation and enhancing PINK1/Parkin mitophagy to inhibit AM pyroptosis and HMGB1 release. These findings provide novel insights for therapeutic strategies for sepsis-associated ARDS.

## Introduction

Sepsis is a complex, multistep pathological process characterized by an excessive inflammatory response of host cells to both Gram-positive and Gram-negative bacteria [1]. The lungs are among the most vulnerable organs during sepsis, with over half of septic patients developing acute respiratory distress syndrome (ARDS) [2]. Accumulating evidence indicates that sepsis can induce programmed cell death in alveolar macrophages (AMs), particularly pyroptosis, which contributes to the progression of ARDS [3]. Therefore, inhibiting or reducing AM pyroptosis may serve as a potential therapeutic strategy to alleviate sepsis-induced ARDS.

Our previous series of investigations revealed that AM pyroptosis and its inflammatory mediator, high mobility group box 1 (HMGB1), induced by cardiopulmonary bypass and sepsis led to the translocation of HMGB1 from the nucleus to the cytoplasm and ultimately to the extracellular fluid, thereby initiating and amplifying the inflammatory response and causing pulmonary damage [ 4, 5]. Thus, suppressing AM pyroptosis and HMGB1 secretion could serve as a therapeutic approach to mitigate ARDS in sepsis. Recently, with respect to the pathogenesis of sepsis, the activation of the Nod-Like Receptor Family Pyrin Domain Containing 3 (NLRP3) inflammasome to induce pyroptosis in AMs has been identified as a causative factor [6]. Briefly, NLRP3 recruits the effector proinflammatory cysteinyl aspartate-specific proteinase-1 (caspase-1) via its adaptor, apoptosis-associated speck-like protein containing a CARD (ASC) complex, followed by the activation of caspase-1 [7]. The Gasdermin D (GSDMD) is cleaved by activated caspase-1 into the N-terminal domain of GSDMD (GSDMD-N), which ultimately leads to cell pyroptosis and the secretion of inflammatory cytokines, including IL-1β [8]. Several recent studies have demonstrated that NLRP3 inflammasome activation leads to the release of proinflammatory cytokines such as IL-1β and HMGB1, which play crucial roles in the pathogenesis of ARDS by promoting inflammation and lung tissue damage. Previous studies showed that in a murine model of ARDS, genetic deletion or pharmacological inhibition of NLRP3 significantly attenuated lung injury and improved survival rates, highlighting the importance of NLRP3 as a potential therapeutic target [ 9, 10]. Other studies reported that NLRP3 activation was associated with increased M1 macrophage polarization and pyroptosis and alveolar-capillary barrier disruption in patients with ARDS, further supporting its role in the disease process [ 11– 13].

Cellular mitophagy is a crucial mechanism for maintaining mitochondrial health by selectively degrading damaged mitochondria through the PTEN-induced kinase 1 (PINK1) and Parkin RBR E3 ubiquitin-protein ligase (PARKIN) pathways [14], which can establish intracellular homeostasis to resist programmed cell death, such as pyroptosis [15]. When the cell is subjected to stimulation by Gram-negative bacteria, specifically LPS, healthy mitochondria fragment and subsequently generate reactive oxygen species (ROS) [16], which induces NLRP3 inflammation-mediated pyroptosis [17]. In mammals, increasing evidence indicates that a pathway involving PINK1 and Parkin participates in mitophagy by eliminating damaged mitochondria to remove ROS in the presence of a loss of the mitochondrial membrane potential [ 18, 19]. NF-E2-related factor 2 (Nrf2), a crucial transcription factor, has been shown to play a pivotal role in promoting mitophagy by upregulating the expression levels of the PINK1 and Parkin proteins [20]. Additionally, NRF2 activation can effectively inhibit ROS- and NLRP3-mediated esophageal squamous cell pyroptosis [21]. Synthetic CDDO-imidazolide (CDDO-Im) is a small-molecule compound and an Nrf2 activator that suppresses the inflammatory response. CDDO-Im is an extremely potent and multifunctional electrophilic Nrf2 activator that is significantly more effective in enhancing Nrf2-dependent cytoprotective gene expression than commonly used Nrf2 activators such as dimethyl fumarate [22]. Xu *et al*. [23] reported that CDDO-Im ameliorated liver injury through the Nrf2 pathway to increase autophagy. Our study was designed primarily to investigate the role of CDDO-Imidazole in Nrf2 activation and the subsequent consequences for cellular processes relevant to our research. Nevertheless, it remains unclear whether CDDO-Im exerts a lung-protective effect in sepsis-associated ARDS by promoting mitophagy via the Nrf2 pathway.

In this study, we aimed to investigate whether CDDO-Im could suppress AM pyroptosis by enhancing mitophagy through the Nrf2 pathway both *in vitro* and *in vivo*.

## Materials and Methods

### Mice, sepsis model, CDDO-Im and Nrf2 inhibitor administration

Male wild-type C57BL/6 mice aged 8–10 weeks were procured from Cyagen Biosciences, Inc. (Shanghai, China). Throughout the study, these mice were cared for in strict accordance with the guidelines stipulated in the National Institutes of Health Guide for the Care and Use of Laboratory Animals. They were maintained in a specific living environment with a 12/12-h light/dark cycle and a temperature range of 22–24°C and had *ad libitum* access to food and water. All experimental procedures involving these mice were subject to review and approved by the Ethics Committee of Shanghai East Hospital, ensuring that the study adhered to ethical standards and animal welfare regulations.

The sepsis model was established using the cecal ligation and puncture (CLP) method, in which approximately 30% of the cecum was ligated, which is consistent with previous reports [24]. Specifically, the mice were first anaesthetized through inhalation of isoflurane. After the abdominal area was disinfected and shaved, a 22-gauge needle was subsequently used to puncture the cecum, allowing a small amount of feces to be extruded. The sham-operated mice (serving as the control group) underwent the same surgical procedures as those in the operation group, with the exception of the caecal ligation and puncture steps.

For the administration of substances, CDDO-Im (No. 443104-02-7; MedchemExpress, Monmouth Junction, USA) at a dose of 30 μmol/kg in a volume of 200 μL, was administered via gavage to the mice 24 h prior to CLP surgery. CDDO-Im was dissolved in DMSO (dimethyl sulfoxide) to prepare a stock solution, which was then further diluted in sterile phosphate-buffered saline (PBS) to the appropriate working concentrations for *in vitro* and *in vivo* experiments. The DMSO concentration in the final working solution was maintained at less than 0.1% to minimize any potential solvent effects on the cells and animals. ML385 (No. 846557-71-9; MedchemExpress), which functions as an inhibitor of Nrf2, was used at a dosage of 30 mg/kg. ML385 was dissolved in a mixture of 0.9% normal saline and DMSO at a ratio of 1:1 (v/v), which means that the DMSO concentration in the solvent used for ML385 was 50% (v/v). This solution was subsequently injected intraperitoneally into septic mice 1 h before CDDO-Im treatment was initiated.

### Cell culture, stimulation, transfection and treatment

The mouse mononuclear macrophage J774A.1 cell line, obtained from the American Type Culture Collection (Manassas, USA), was cultured in Dulbecco’s modified Eagle’s medium (DMEM; HyClone, Logan, USA). The culture medium was supplemented with 10% fetal bovine serum, 100 U/mL penicillin, and 100 μg/mL streptomycin (Thermo Fisher Scientific, Waltham, USA). The cells were maintained in a 37°C incubator with an atmosphere composed of 95% air and 5% CO _2_.

To establish an *in vitro* pyroptosis model, J774A.1 cells were first stimulated with 1 μg/mL lipopolysaccharide (LPS; Sigma-Aldrich, St Louis, USA) for 4 h. Subsequently, adenosine triphosphate (ATP; Sigma-Aldrich) was added at a final concentration of 5 mM to the cell culture for an additional 30 min.

To investigate the role of Nrf2 in the regulation of pyroptosis, J774A.1 cells were transfected with a lentivirus designed to knockdown *Nrf2*, along with a control scramble lentivirus (designated control-KO). Both types of lentiviruses were designed by Wsan Biotechnology (No. WS-SH20078; Shanghai, China).

To clarify the role of mitophagy in regulating macrophage pyroptosis, J774A.1 cells were pretreated with 5 mM of 3-methyladenine (3MA; Sigma-Aldrich), a mitophagy inhibitor, for 3 h prior to being stimulated with LPS and ATP. To explore the role of CDDO-Im in regulating the Nrf2 pathway, both *Nrf2*-knockout ( *Nrf2*-KO) and control-KO J774A.1 cells were pretreated with CDDO-Im at increasing concentrations of 10 nM, 50 nM, and 100 nM for 3 h before being stimulated with LPS and ATP.

### Cellular ROS detection

The intracellular levels of reactive oxygen species (ROS) were detected using 2′,7′-dichlorofluorescein diacetate (DCFH-DA; Beyotime Biotechnology, Shanghai, China). To prepare the working solution, DCFH-DA was diluted in serum-free DMEM at a dilution ratio of 1:1000, resulting in a final concentration of 10 μM.

The collected cells were then resuspended in 500 μL of the diluted DCFH-DA solution and subsequently incubated at 37°C for 30 min. After incubation, the cells were washed three times with serum-free DMEM to remove any residual unreacted DCFH-DA.

Finally, the intracellular ROS levels were determined with a flow cytometer (Olympus, Tokyo, Japan) with an excitation wavelength of 504 nm and an emission wavelength of 529 nm.

### ASC oligomerization detection

ASC oligomerization was detected as previously reported [25]. Briefly, the collected cell pellets were first resuspended in specialized buffer composed of 20 mM HEPES-KOH (pH 7.5), 150 mM KCl, 1% Nonidet P-40, and 0.1 mM PMSF. A protease inhibitor was added to this buffer to prevent protein degradation during the subsequent steps. The cells were subsequently lysed by passing them through a 21-gauge needle multiple times to disrupt the cell membranes and release the intracellular contents.

Next, the lysed samples were centrifuged at 5000 *g* for 10 min at 4°C. The resulting supernatants were then collected and crosslinked with 4 mM disuccinimidyl suberate (DSS) for 30 min at 37°C. This cross-linking step is crucial for stabilizing the ASC oligomers.

Finally, the crosslinked samples were added to SDS buffer and boiled for a certain period to denature the proteins. The samples were subsequently separated by 12% sodium dodecyl–sulphate polyacrylamide gel electrophoresis (SDS-PAGE). After electrophoresis, the proteins were transferred onto a membrane and immunoblotted with an ASC rabbit monoclonal antibody (specific for mouse; #67824; Cell Signaling Technology, Danvers, USA). This immunoblotting step enabled the visualization and quantification of the ASC oligomers on the basis of the antibody’s binding specificity.

### Western blot analysis

Total proteins were extracted from both lung tissues and cell lysates. The samples were subsequently centrifuged at 20,000 *g* for 10 min. The protein concentrations in the supernatants were then determined via a bicinchoninic acid (BCA) protein assay kit (Thermo Fisher Scientific). Equal amounts of lysate samples (50 μg per lane) were separated via sodium dodecyl sulfate-polyacrylamide gel electrophoresis (SDS-PAGE) on 10%–15% gels (Beyotime Biotechnology). After electrophoresis, the proteins were transferred onto polyvinylidene difluoride (PVDF) membranes.

The PVDF membranes were blocked with 5% bovine serum albumin (BSA) in Tris-buffered saline (TBST) for 1 h at room temperature. The membranes were subsequently incubated overnight with the appropriate primary antibodies. Following this incubation, the membranes were thoroughly washed and then incubated with horseradish peroxidase (HRP)-conjugated secondary antibodies for 1 h. Finally, the protein bands were detected by chemiluminescence using the appropriate detection system (Bio-Rad Laboratories, Inc., Hercules, USA).

The primary antibodies used in this study were as follows: anti-HMGB1 (diluted at a ratio of 1:1000; ab79823; Abcam, Cambridge, UK), anti-pro-caspase-1 + p10 (diluted 1:1000, ab179515; Abcam), anti-ASC (diluted 1:1000; #67824; Cell Signaling Technology), anti-NLRP3 (diluted 1:1000; #15101; Cell Signaling Technology), anti-GSDMD (diluted 1:1000; ab219800; Abcam), anti-PINK1 (diluted 1:1000; ab23707; Abcam), anti-Parkin (diluted 1:1000; ab77924; Abcam), anti-β-actin (diluted 1:1000; #4970; Cell Signaling Technology), and anti-LC3B (diluted 1:1000; ab192890; Abcam).

### Detection of the HMGB1 protein in the cell supernatant, BALF and serum

The cell supernatants, sera, and bronchoalveolar lavage fluid (BALF) from different groups were collected. These samples were subsequently mixed with 100% trichloroacetic acid at a dilution ratio of 1 mL:100 μL and then incubated at 4°C for 30 min. Afterwards, the mixtures were centrifuged at 15,000–22,000 *g* and 4°C for 5 min. The resulting pellets were then added to ice-cold acetone to remove any residual trichloroacetic acid.

Finally, the pellets were resuspended in loading buffer and boiled at 100°C for 5 min. The protein levels of HMGB1 in these samples were determined by western blot analysis, with Ponceau S used as a positive control.

### Ponceau S staining

The total composition of the transferred proteins can be identified by staining the PVDF membrane with Ponceau S. The following steps were carried out. First, the PVDF membrane was rinsed thoroughly with ultrapure water to remove any impurities. The mixture was subsequently incubated with freshly diluted Ponceau S solution at room temperature for 5 min while being gently rocked to ensure even staining. After that, the Ponceau S solution was removed by quickly rinsing the membrane with water and then with immunoblot wash buffer for 1–2 min. This step aimed to wash away the excess staining solution. Finally, the positions of the transferred proteins as well as the molecular weight markers were marked as needed, which facilitated the subsequent analysis and interpretation of the immunoblotting results.

### Macrophage pyroptosis analysis

After stimulation, the collected cells in different groups were stained with an Alexa Fluor 488-labelled anti-caspase-1 antibody at 37°C for 1 h and then dyed with propidium iodide (PI) for 5 min at room temperature according to the instructions of FAM-FLICA Caspase Assay kit (ImmunoChemistry Technology, Bloomington, USA). The percentages of caspase-1 plus PI pyroptotic macrophages were analyzed via a flow cytometer (Cytoflex B4-R3-V3; Beckman, Pasadena, USA).

### Immunofluorescence staining

Immunofluorescence staining was carried out following the procedure we previously described [4]. Briefly, J774A.1 cells from different groups were first fixed with 4% paraformaldehyde for 30 min. The cells were subsequently permeabilized with 0.1% Triton-100 for another 30 min to enhance the penetration of the antibodies. Then, the sections were blocked with 10% goat serum (Beyotime Biotechnology) for 2 h to reduce non-specific binding of the antibodies. After being washed three times with an appropriate buffer (such as PBS), the cells were incubated with antibodies against LC3 (diluted at a ratio of 1:1000; ab192890; Abcam) and MitoTracker Green FM (Invitrogen, Carlsbad, USA) overnight at 4°C. The following day, the cells were incubated with FITC-labelled goat anti-rabbit IgG (Beyotime Biotechnology) at room temperature for 1 h. Additionally, 4′,6-diamidino-2-phenylindole (DAPI; Santa Cruz Biotech, Santa Cruz, USA) was used for nuclear staining. Finally, mitophagy, which was indicated by the co-localization of LC3B with MitoTracker, was evaluated under an SP8 confocal microscope (Leica, Wetzlar, Germany). This allowed visualization and analysis of the mitophagy process on the basis of the fluorescence signals and their spatial distribution.

### Hematoxylin and Eosin (H&E) staining

The harvested pulmonary tissue was first fixed in 10% buffered formalin to preserve its morphological structure. The fixed tissue was subsequently embedded in paraffin wax following standard procedures. Once embedded, the tissue block was sectioned into slices with a thickness of 5 μm via a microtome. Finally, the obtained sections of the lung tissues were subjected to H&E staining, which enabled the visualization of histological features for subsequent microscopic examination and analysis. Lung tissue damage was scored according to our previous study [26].

### Statistical analysis

Data are presented as the mean ± standard deviation. To compare different groups, one-way analysis of variance (ANOVA) was employed, followed by post hoc Tukey’s multiple comparison test. A two-sided *P* value less than 0.05 was considered statistically significant, indicating that the observed differences between groups are unlikely to have occurred by chance and thus suggest a real effect or difference in the underlying populations.

## Results

### LPS + ATP-individually induces J774A.1 macrophage pyroptosis and slight mitophagy

**Figure 1:**
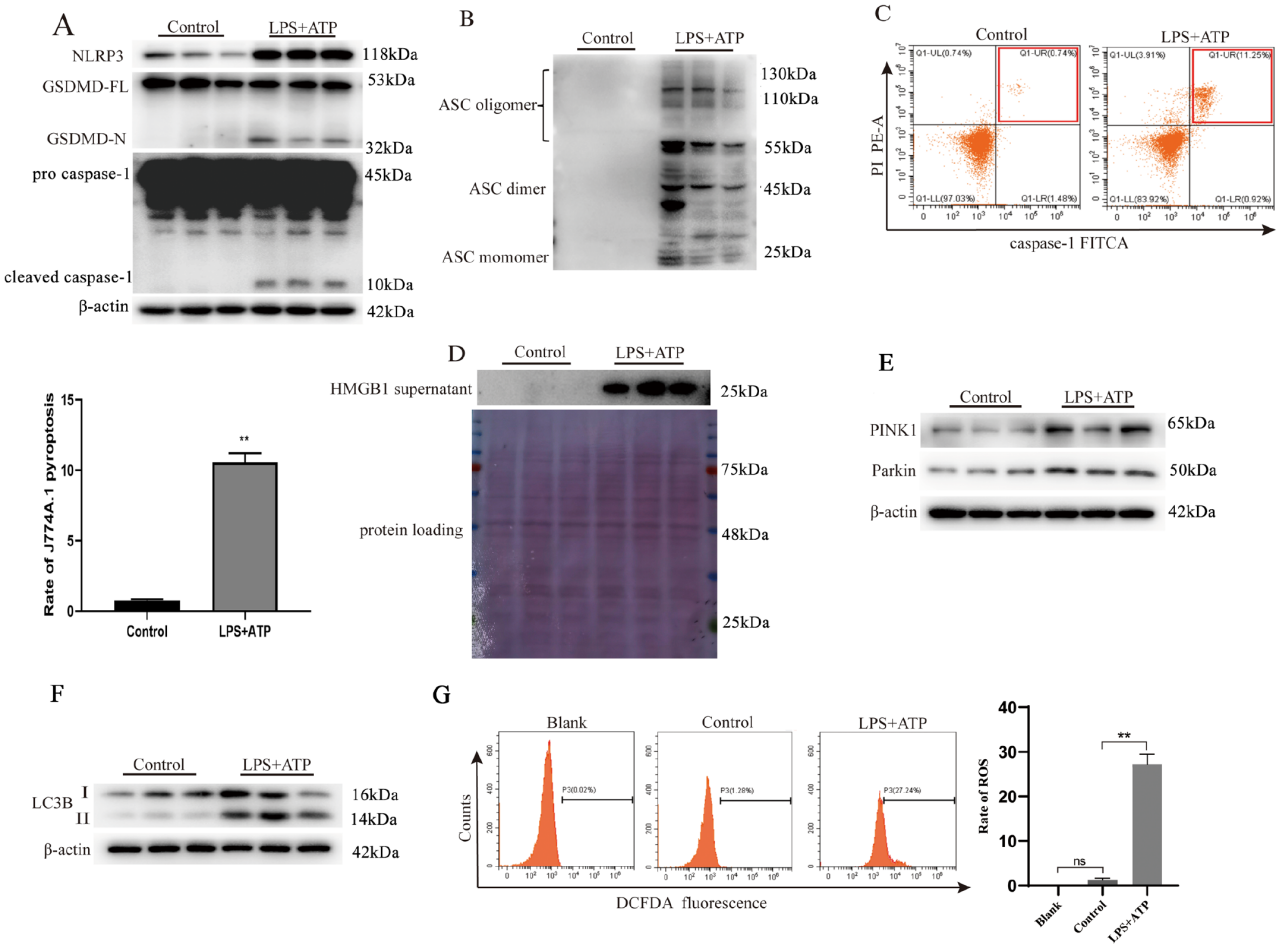
Figure 1 LPS + ATP individually induces pyroptosis and slight mitophagy in J774A.1 macrophages J774A.1 cells were first stimulated with 1 μg/mL LPS for 4 h, followed by treatment with 5 mM ATP for 30 min. (A) Western blot images showing that LPS + ATP treatment successfully promoted the protein expressions of NLRP3, caspase-1 p10 and GSDMD-N. (B) Western blot analysis demonstrating elevated ASC oligomers in LPS + ATP-treated J774A.1 cells. (C) Flow cytometry analysis showing the macrophage pyroptosis ratio. (D) Western blot analysis showing the expression of HMGB1 in the supernatant. LPS + ATP slightly activated PINK1/Parkin-dependent mitophagy and produced abundant ROS in AMs. (E,F) LPS + ATP slightly promoted the protein expressions of PINK1, Parkin and the LC3-II/LC3-I ratio. (G) Cellular ROS levels measured by flow cytometry. Data are expressed as the mean ± SD (n = 3). **P < 0.01 indicates a significant difference from each group, and ns indicates no significance.

To investigate whether LPS combined with ATP (LPS + ATP) induces J774A.1 macrophage pyroptosis through the canonical pathway, western blot analysis was performed. The results demonstrated that LPS + ATP significantly increased the protein levels of NLRP3, caspase-1 p10, and GSDMD-N in the macrophage lysates ( Figure 1A). Notably, the ASC oligomer level was also substantially elevated in the J774A.1 lysates upon LPS + ATP treatment ( Figure 1B). Flow cytometry analysis revealed that the J774A.1 macrophage pyroptosis ratio in the LPS + ATP group was approximately 11% greater than that in the control group (untreated J774A.1 cells) ( Figure 1C). Moreover, western blot analysis revealed that the HMGB1 protein level in the cell supernatant, which reflects macrophage pyroptosis and inflammatory cytokine secretion, was greater in the LPS + ATP treatment group than in the control group ( Figure 1D). Overall, LPS + ATP can undoubtedly induce AM pyroptosis and HMGB1 secretion. In general, mitophagy involving the PINK1/Parkin-mediated ubiquitin-dependent mitophagy pathway has been extensively studied in various cell models [27]. After ubiquitination and mitophagy, the LC3-I protein is converted into the autophagic vesicle form (LC3-II), which serves as an important marker of intracellular autophagic activity [28]. Our results revealed that LPS + ATP treatment slightly increased the levels of PINK1, Parkin, and the LC3-II/LC3-I ratio in J774A.1 macrophage lysates ( Figure 1E,F). Nevertheless, a significant amount of ROS could still be detected by flow cytometry following LPS + ATP treatment ( Figure 1G,H), suggesting that the slightly activated mitophagy is insufficient to provide adequate protection against J774A.1 ROS and pyroptosis.

### The inhibition of PINK1/Parkin-dependent mitophagy contributes to increased ROS production, which aggravates J774A.1 macrophage pyroptosis

**Figure 2:**
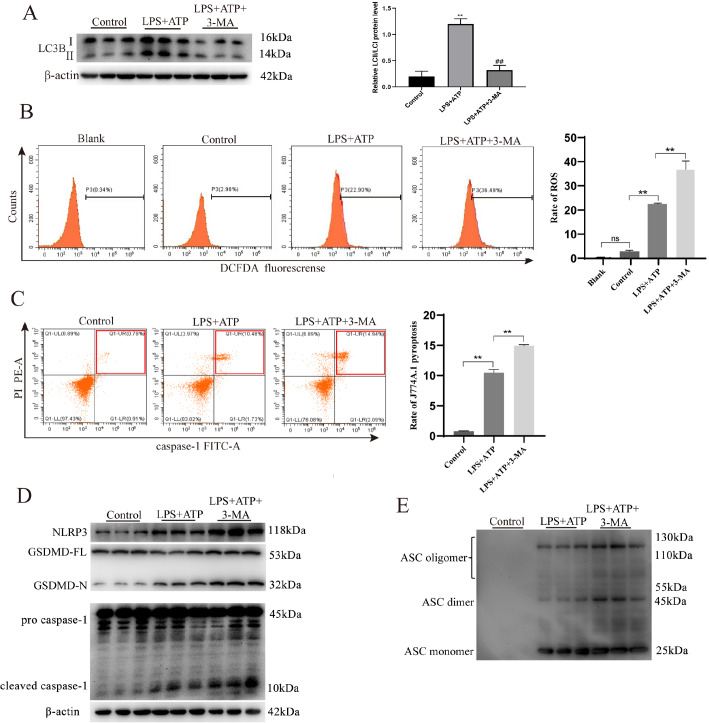
Figure 2 The inhibition of PINK1/Parkin-dependent mitophagy contributes to increased ROS production, which aggravates J774A.1 macrophage pyroptosis J774A.1 cells were pretreated with the mitophagy inhibitor 3-MA (5 mM) for 3 h and then co-cultured with LPS and ATP. (A) 3-MA reduced LC3-II/LC3-I protein expression, as indicated by western blot analysis. (B) 3-MA promoted ROS production in the LPS + ATP-treated J774A.1 cells. (C) 3-MA promoted macrophage pyroptosis, as indicated by flow cytometry, in the LPS + ATP-treated J774A.1 cells. (D) Western blot analysis showing increased protein expressions of NLRP3, caspase-1 p10 and GSDMD-N in the LPS + ATP-treated J774A.1 cells. (E) 3-MA increased the ASC oligomer levels in the LPS + ATP-treated J774A.1 cells. Data are expressed as the mean ± SD (n = 3). **P < 0.01 indicates significant differences from each group, ##P < 0.01 vs the LPS+ATP group, and ns indicates no significant difference.

According to previous studies [ 29, 30], 3-MA, which serves as an autophagy inhibitor, significantly reduces the protein levels of PINK1 and Parkin. These findings indicate that 3-MA can also function as a mitophagy inhibitor to suppress PINK1/Parkin-dependent mitophagy in J774A.1 cells. In our study, to assess the relationship between mitophagy and pyroptosis, J774A.1 macrophages were pretreated with 3-MA prior to co-culture with LPS and ATP. Western blot analysis demonstrated that 3-MA effectively prevented the increase in LC3-II/LC3-I protein levels in J774A.1 macrophages treated with LPS + ATP ( Figure 2A). Additionally, we detected greater generation of excessive ROS in J774A.1 macrophages pretreated with 3-MA after exposure to LPS + ATP ( Figure 2B). These results suggest that the inhibition of PINK1/Parkin-dependent mitophagy contributes to the accumulation of ROS in LPS + ATP-treated J774A.1 macrophages. We subsequently explored the relationship between mitophagy and pyroptosis. Both flow cytometry and western blot analysis results indicated that 3-MA promoted significant macrophage pyroptosis. This was evidenced by an increased proportion of caspase-1 and PI double-positive cells, as well as elevated protein levels of NLRP3, caspase-1 p10, GSDMD-N, and ASC oligomers ( Figure 2C–E). Therefore, the blockade of PINK1/Parkin-dependent mitophagy contributes to increased ROS production, which in turn exacerbates J774A.1 macrophage pyroptosis.

### CDDO-Im promotes PINK1/Parkin-dependent mitophagy to suppress J774A.1 macrophage pyroptosis and HMGB1 release via the Nrf2 pathway

**Figure 3:**
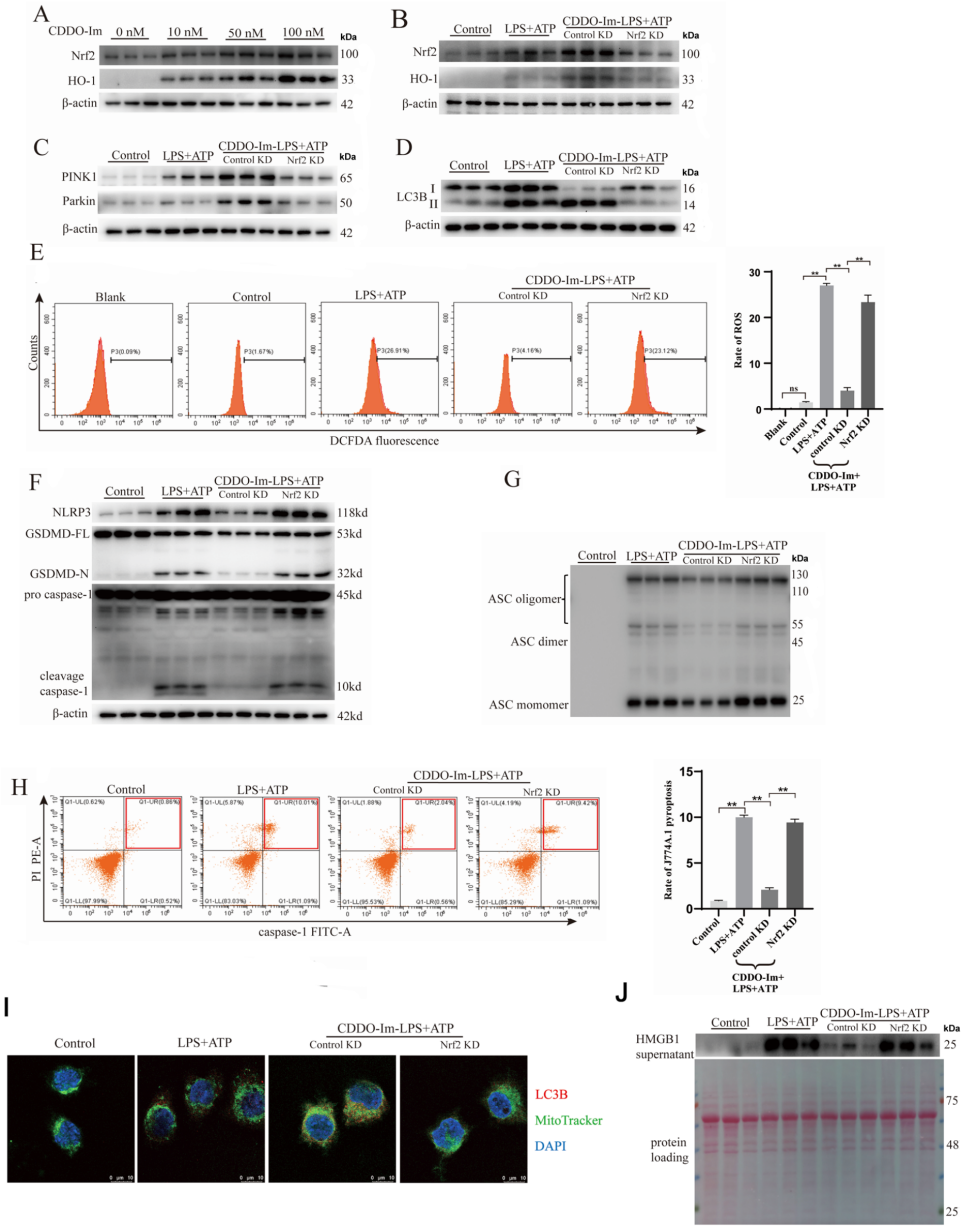
Figure 3 CDDO-Im promotes PINK1/Parkin-dependent mitophagy to suppress J774A.1 macrophage pyroptosis and HMGB1 release via the Nrf2 pathway Nrf2-KO and control-KO J774A.1 cells were pretreated with 10 nM, 50 nM or 100 nM CDDO-Im for 3 h before being exposed to LPS and ATP. (A) Western blot analysis revealed that 100 nM CDDO-Im significantly promoted Nrf2 and HO-1 protein expressions in LPS + ATP-treated J774A.1 cells. (B) CDDO-Im failed to promote the expressions of Nrf2 and HO-1 in the LPS + ATP-treated Nrf2 (–/–) J774A.1 cells. (C) The protein expressions of PINK and Parkin in Nrf2 (–/–) J774A.1 cells treated with LPS+ATP were inhibited even in the presence of CDDO-Im. (D) CDDO-Im was unable to promote mitophagy in the LPS + ATP-treated Nrf2 (–/–) J774A.1 cells. (E) CDDO-Im did not affect the cellular ROS levels in the LPS + ATP-treated Nrf2 (–/–) J774A.1 cells. (F,G) Western blot analysis showing that CDDO-Im failed to reduce the levels of NLRP3, caspase-1 p10, GSDMD-N and ASC oligomers in Nrf2 (–/–) J774A.1 cells treated with LPS + ATP. (H,J) Inability of CDDO-Im to prohibit pyroptosis and the release of HMGB1 in LPS+ATP-treated Nrf2 (–/–) J774A.1 cells. (I) CDDO-Im failed to promote the colocalization of LCB and MitoTracker in the LPS + ATP-treated Nrf2 (–/–) J774A.1 cells. Data are expressed as the mean ± SD (n = 3). **P < 0.01 indicates a significant difference from each group, and ns indicates no significance.

To determine whether CDDO-Im promotes mitophagy to suppress J774A.1 macrophage pyroptosis by activating the Nrf2 pathway, J774A.1 cells were first pretreated with different concentrations of CDDO-Im (10 nM, 50 nM, or 100 nM) before being cocultured with LPS plus ATP. CDDO-Im at a concentration of 100 nM significantly enhanced Nrf2 pathway activation in the LPS plus ATP-treated J774A.1 cells, as evidenced by elevated protein levels of Nrf2 and HO-1 ( Figure 3A). Following knockdown of the *Nrf2* gene using lentivirus, this phenomenon was no longer detected ( Figure 3B). Furthermore, western blot analysis revealed that CDDO-Im was unable to increase the levels of mitophagy-related proteins, including PINK1, Parkin, and LC3-II/LC3-I, indicating that CDDO-Im failed to promote mitophagy in LPS plus ATP-treated *Nrf2*-knockout [Nrf2 (–/–)] J774A.1 macrophages ( Figure 3C,D). Immunofluorescence staining revealed that CDDO-im could not promote the colocalization of LCB and mitochondria, indicating that CDDO-Im was unable to promote mitophagy in LPS plus ATP-treated Nrf2 (–/–) J774A.1 macrophages ( Figure 3I). Moreover, the ability of CDDO-Im to reduce ROS levels ( Figure 3E), prevent NLRP3 inflammasome activation-mediated J774A.1 pyroptosis ( Figure 3F–H), and suppress HMGB1 release ( Figure 3J) in LPS plus ATP-treated Nrf2 (–/–) J774A.1 cells was abolished. Therefore, CDDO-Im promotes PINK1/Parkin-dependent mitophagy to suppress J774A.1 macrophage pyroptosis and HMGB1 release via the Nrf2 pathway.

### CDDO-Im alleviates NLRP3 inflammasome activation and HMGB1 release through the Nrf2 pathway to activate mitophagy in sepsis-related ARDS

**Figure 4:**
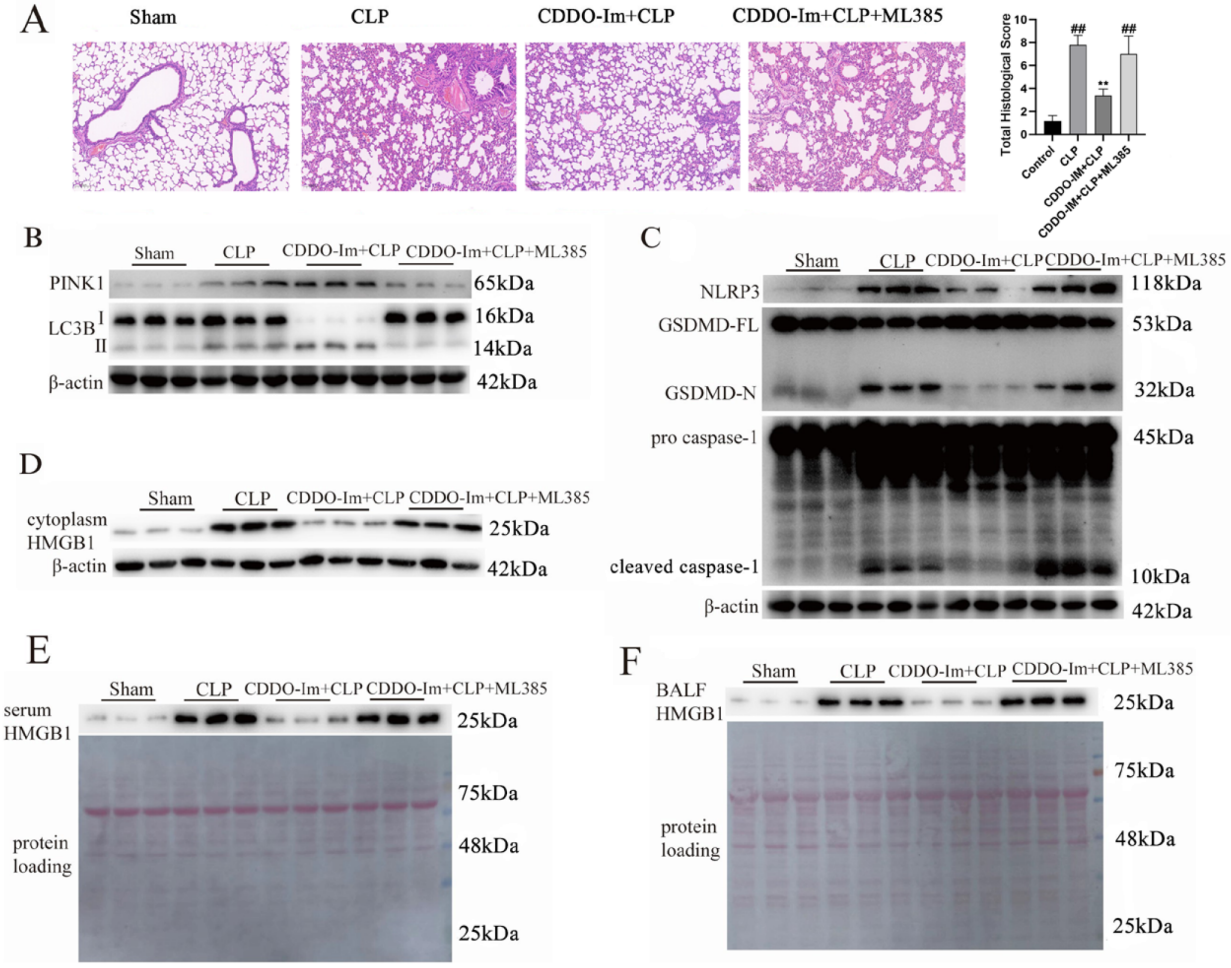
Figure 4 CDDO-Im alleviates NLRP3 inflammasome activation and HMGB1 release through the Nrf2 pathway to activate mitophagy in sepsis-related ARDS The mice were injected intraperitoneally with ML385 (30 mg/kg) 1 h before they were treated with CDDO-Im (30 μmol/kg in 200 μL volume) for another 23 h, after which the mice were subjected to the caecal ligation puncture procedure. (A) Pathologic examination (magnification 100×) and histologic scoring revealed that, compared with those in the control group, the lungs of the mice that underwent CLP surgery were severely damaged, as indicated by the amount of inflammatory cell infiltration and widening of the alveolar septa. Nonetheless, after pretreatment with CDDO-Im, the damage to the lung tissue was alleviated, with less inflammatory cell infiltration and widening of the alveolar septa. The protective effect of CDDO-Im was abolished by ML385, as indicated by the severe lung damage shown in the CDDO-Im + CLP + ML385 group. (B) CDDO-Im promoted PINK1 and LC3BII protein expressions in the lung tissue following sepsis exposure in wild-type C57BL/6 mice. (C) CDDO-Im inhibited NLRP3, cleaved caspase-1 p10 and GSDMD-N protein expressions in lung tissue following sepsis exposure in wild-type C57BL/6 mice. (D-F) Western blot analysis showing reduced HMGB1 protein levels in lung tissue, serum and BALF following sepsis exposure in wild-type C57BL/6 mice subjected to CDDO-Im treatment. However, the above role of CDDO-Im was not observed in ML385-treated mice. ##P < 0.01 vs the control group, **P < 0.01 vs the CLP group.

To investigate the protective role of CDDO-Im in ARDS and its underlying mechanism, the Nrf2 inhibitor ML385 was administered 1 h prior to treatment with CDDO-Im in septic mice. Pathologic examinations and histologic scoring revealed that CDDO-Im significantly reduced lung damage resulting from sepsis exposure. Specifically, it improved alveolar septal thickening, interstitial edema, vascular congestion, and interstitial neutrophil infiltration in the lung tissues of septic mice and decreased the total histologic score ( Figure 4A). Next, we evaluated whether the effects of CDDO-Im treatment in a mouse model of sepsis-related ARDS are consistent with those observed in cultured J774A.1 macrophages. CDDO-Im treatment also promoted the expression of mitophagy-related proteins, such as PINK1 and LC3-II/LC3-I, and inhibited the expressions of pyroptosis-related proteins, including NLRP3, cleaved caspase-1 p10, and GSDMD-N, in the lung tissues of septic mice ( Figure 4B,C). However, CDDO-Im was unable to regulate the expressions of the aforementioned proteins in the lung tissues of septic mice pretreated with ML385 ( Figure 4B,C). These results suggest that CDDO-Im activates mitophagy to suppress NLRP3 inflammasome-mediated caspase-1 activation in the lung tissues of septic mice. The levels of HMGB1 in the serum and BALF reflect the release of inflammatory factors due to alveolar macrophage pyroptosis, which contributes to lung damage [25]. We initially found that CDDO-Im significantly reduced HMGB1 protein levels in the cytoplasm of lung tissue following sepsis exposure ( Figure 4D). Most importantly, CDDO-Im also decreased HMGB1 protein levels in the serum and BALF after sepsis exposure ( Figure 4E,F). However, CDDO-Im was unable to reduce HMGB1 levels in septic mice treated with ML385. Overall, CDDO-Im alleviates NLRP3 inflammasome activation and HMGB1 release through the Nrf2 pathway, thereby activating mitophagy in sepsis-related ARDS.

## Discussion

Previous studies have demonstrated the ability of CDDO-Im to exert tissue protection, for example, in liver and kidney ischaemia‒reperfusion injury [ 23, 31]. Notably, whether CDDO-Im can also alleviate sepsis-associated ARDS and its underlying mechanism have not yet been investigated. In the present study, we are the first to report the protective role of CDDO-Im against sepsis-induced ARDS by maintaining a delicate balance between mitophagy and pyroptosis. Unlike the findings of Thomas, where CDDO-Im significantly reduced lung oxidative stress and alveolar epithelial cell apoptosis [32], our findings initially indicate that CDDO-Im can reduce alveolar macrophage pyroptosis and HMGB1 release by promoting mitophagy in both *in vivo* and *in vitro* sepsis-treated models. The previously observed trend of changes in inflammatory responses in lung tissue corresponds with known AM-related inflammatory pathways, especially NLRP3 inflammasome activation-mediated pyroptosis [33]. More targeted experiments, such as specific labelling and tracking of AMs to further explore their dynamic changes and mechanism of action in the CDDO-Im-mediated process, may be needed in the future. Our results highlight that the activation of PINK1/Parkin-mediated mitophagy to suppress pyroptosis via the Nrf2 pathway may serve as a potential therapeutic target for CDDO-Im in alleviating sepsis-induced ARDS.

PINK1 and Parkin physically interact and functionally collaborate to eliminate damaged mitochondria through ubiquitin-dependent mitophagy [34]. Specifically, under hypoxia-reoxygenation stress or LPS stimulation, PINK1 translocates from the inner mitochondrial membrane to the outer mitochondrial membrane. Subsequently, it selectively recruits cytosolic Parkin to depolarized mitochondria, thereby initiating autophagic flux [ 35– 37]; as we demonstrated in the present study, only slight activation of PINK1/Parkin-dependent mitophagy was observed in J774A.1 cells treated with LPS and ATP. Many previous studies have confirmed that PINK1-Parkin-mediated mitophagy can effectively reduce mtROS and inhibit the subsequent activation of the NLRP3 inflammasome under a variety of medical conditions [ 38, 39]. However, the slightly elevated PINK1/Parkin-mediated mitophagy was insufficient to suppress cellular mtROS production and J774A.1 pyroptosis in AMs treated with LPS and ATP. Moreover, an earlier study reported that the release of mitochondrial DNA triggered by PINK1-dependent mitophagy aggravated stretch-induced lung inflammation [40]. This aggravation is achieved by activating NLRP3-mediated pyroptosis in the context of LPS-induced ARDS [41]. It remains unclear whether mitophagy activation can alleviate NLRP3-mediated pyroptosis in septic alveolar macrophages. In our study, the mitophagy-specific inhibitor 3-MA was employed, and it was found to aggravate cellular ROS production, pyroptosis, and HMGB1 secretion in alveolar macrophages after LPS and ATP stimulation. This finding is consistent with other studies showing that 3-MA inhibits mitophagy, causes mitochondrial damage, and promotes NLRP3-mediated pyroptosis [ 42, 43]. It is clear that PINK1/Parkin-dependent mitophagy, which serves as a mechanism for mitochondrial quality control to eliminate abundant ROS, may resist NLRP3-mediated pyroptosis and HMGB1 release under LPS + ATP stimulation. In addition, the activation of PINK1-mediated mitophagy has been reported to protect against cecal ligation and puncture (CLP)-induced ALI [44]. PINK1- and Parkin-deficient mice are more sensitive to polymicrobial sepsis-induced multiple organ failure and death [45]. 3-MA exerts a protective effect on lung ischaemia-reperfusion injury [46]. PINK1/Parkin-mediated mitophagy protects against kidney damage in mice [47]. These results indicate that enhancing mitophagy may be a potential therapeutic strategy to alleviate alveolar macrophage pyroptosis in CLP-induced ARDS.

Experimental evidence has confirmed that the activated Nrf2 pathway is involved in restoring impaired mitophagy by promoting PINK1 gene expression [ 48, 49]. Consistent with the findings reported in RAW264.7 macrophage-like cells by Cao *et al*. [50], our study demonstrated that CDDO-Im promoted the protein expressions of Nrf2 and its downstream target, HO-1, in a dose-dependent manner. Specifically, the expressions of Nrf2 and HO-1 were significantly increased at a CDDO-Im concentration of 100 nM, and this effect could be reversed by *Nrf2* gene knockout in alveolar macrophages. Previous studies have shown that NLRP3 inflammasome activation-mediated pyroptosis is dependent on ROS [ 51, 52]. Correspondingly, our study revealed that CDDO-Im significantly initiated PINK1/Parkin-dependent mitophagy and autophagic flux through the Nrf2 pathway, thereby removing excessive ROS and further reducing NLRP3 inflammasome-induced alveolar macrophage pyroptosis. Previous reports have shown that pharmacological manipulation of Nrf2 pathway activation can alleviate ARDS by inhibiting ROS production and NLRP3 inflammasome-mediated pyroptosis [53]. In our study, we further found that *in vitro* *Nrf2* knockdown or *in vivo* inhibition of Nrf2 expression in mice using ML385 led to the inability of CDDO-Im to promote PINK1/Parkin-dependent mitophagy to prevent AM pyroptosis and HMGB1 release. Most importantly, CDDO-Im alleviated sepsis-associated ARDS, as evidenced by improvements in alveolar septal thickening, interstitial edema, and HMGB1 expression in BALF. However, this phenomenon could be reversed by the Nrf2 inhibitor ML385.

HMGB1 is widely recognized as a key player in various inflammatory processes, particularly in the context of lung injury [54]. It exists in the nucleus of most cells under normal physiological conditions, where it plays roles in DNA binding, chromatin remodeling, and transcriptional regulation [55]. However, during tissue injury, necrosis, or inflammation, HMGB1 can be released from the nucleus into the extracellular space, where it exerts proinflammatory effects [56]. In the context of lung injury, several mechanisms have been proposed for the contribution of HMGB1. First, extracellular HMGB1 can act as a damage-associated molecular pattern (DAMP) molecule that binds to pattern recognition receptors (PRRs), such as Toll-like receptors (TLRs) and receptors for advanced glycation end products (RAGE), on immune cells, including macrophages, neutrophils, and dendritic cells [57]. The activation of these receptors leads to the initiation of downstream signaling cascades, resulting in the production of proinflammatory cytokines such as TNF-α, IL-1β, and IL-6. For example, a previous study showed that HMGB1-TLR4 interaction in lung macrophages promotes the release of TNF-α, thereby exacerbating the inflammatory response and contributing to lung inflammation and subsequent tissue damage [58]. Moreover, blocking HMGB1 reduced neutrophil recruitment and attenuated lung injury in a murine model of acute lung injury, highlighting the importance of HMGB1 in neutrophil-mediated tissue damage [59]. In addition, HMGB1 can regulate vascular permeability in the lungs. By affecting endothelial cell junctions, it promotes the leakage of proteins and fluid from capillaries into the alveolar space, worsening pulmonary edema [60]. Another aspect of the role of HMGB1 in lung injury is its interaction with other inflammatory pathways. This interaction is mediated through the interaction of HMGB1 with various components of the NLRP3 inflammasome, and its activation can lead to pyroptosis, a form of programmed cell death associated with inflammation [61]. Huang *et al*. [62] reported that HMGB1-induced NLRP3 inflammasome activation contributes to the development of acute lung injury in sepsis-induced models. Extracellular HMGB1 can induce NLRP3-mediated AM pyroptosis through its receptor TLR4 signaling pathway in LPS-induced ARDS [63]. In our study, we observed changes in HMGB1 levels under different experimental conditions ( *e*. *g*., in cell supernatants, BALF, and serum), which may indicate its involvement in the pathogenesis of lung injury. Additionally, inhibition of the NLRP3 pathway via an Nrf2 activator attenuated the release of HMGB1. This finding is consistent with previous findings that the activation of Nrf2 by certain small molecule drugs could suppress HMGB1 [ 64, 65]. Moreover, the inhibition of HMGB1/RAGE axis activation led to an increase in stress-induced mitophagy flux [66]. On the basis of these observations, it is hypothesized that CDDO-Im could further promote mitophagy within alveolar macrophages while simultaneously inhibiting pyroptosis and the secretion of HMGB1. Overall, the present study reveals for the first time that CDDO-Im can successfully inhibit the release of HMGB1 in the supernatant, serum, and BALF by blocking alveolar macrophage pyroptosis. However, it is worth noting that this inhibitory effect disappears in the presence of the Nrf2 inhibitor ML385. Taken together, these results support the conclusion that CDDO-Im activates Nrf2 and that activated Nrf2, in turn, inhibits the HMGB1 release mediated by alveolar macrophage pyroptosis.

In conclusion, we demonstrated that CDDO-Im alleviates sepsis-related ARDS through the activation of PINK1/Parkin-mediated mitophagy to inhibit AM pyroptosis and HMGB1 release both *in vivo* and *in vitro* via the Nrf2 pathway. Additional studies are needed to explore the translational potential of CDDO-Im for preventing lung damage in clinical practice.
